# Isolation and genome-wide expression and methylation characterization of CD31^+^ cells from normal and malignant human prostate tissue

**DOI:** 10.18632/oncotarget.1269

**Published:** 2013-08-21

**Authors:** Wei Luo, Qiang Hu, Dan Wang, Kristin K. Deeb, Yingyu Ma, Carl D. Morrison, Song Liu, Candace S. Johnson, Donald L. Trump

**Affiliations:** ^1^ Department of Pharmacology and Therapeutics, Roswell Park Cancer Institute, Buffalo, New York; ^2^ Department of Pathology, Roswell Park Cancer Institute, Buffalo, New York; ^3^ Department of Biostatistics and Bioinformatics, Roswell Park Cancer Institute, Buffalo, New York; ^4^ Department of Medicine, Roswell Park Cancer Institute, Buffalo, New York

**Keywords:** Endothelial cells, Gene expression, DNA methylation, prostate cancer

## Abstract

Endothelial cells (ECs) are an important component involved in the angiogenesis. Little is known about the global gene expression and epigenetic regulation in tumor endothelial cells. The identification of gene expression and epigenetic difference between human prostate tumor-derived endothelial cells (TdECs) and those in normal tissues may uncover unique biological features of TdEC and facilitate the discovery of new anti-angiogenic targets. We established a method for isolation of CD31^+^ endothelial cells from malignant and normal prostate tissues obtained at prostatectomy. TdECs and normal-derived ECs (NdECs) showed >90% enrichment in primary culture and demonstrated microvascular endothelial cell characteristics such as cobblestone morphology in monolayer culture, diI-acetyl-LDL uptake and capillary-tube like formation in Matrigel^®^. *In vitro* primary cultures of ECs maintained expression of endothelial markers such as CD31, von Willebrand factor, intercellular adhesion molecule, vascular endothelial growth factor receptor 1, and vascular endothelial growth factor receptor 2. We then conducted a pilot study of transcriptome and methylome analysis of TdECs and matched NdECs from patients with prostate cancer. We observed a wide spectrum of differences in gene expression and methylation patterns in endothelial cells, between malignant and normal prostate tissues. Array-based expression and methylation data were validated by qRT-PCR and bisulfite DNA pyrosequencing. Further analysis of transcriptome and methylome data revealed a number of differentially expressed genes with loci whose methylation change is accompanied by an inverse change in gene expression. Our study demonstrates the feasibility of isolation of ECs from histologically normal prostate and prostate cancer via CD31^+^ selection. The data, although preliminary, indicates that there exist widespread differences in methylation and transcription between TdECs and NdECs. Interestingly, only a small proportion of perturbed genes were overlapped between American (AA) and Caucasian American (CA) patients with prostate cancer. Our study indicates that identifying gene expression and/or epigenetic differences between TdECs and NdECs may provide us with new anti-angiogenic targets. Future studies will be required to further characterize the isolated ECs and determine the biological features that can be exploited in the prognosis and therapy of prostate cancer.

## INTRODUCTION

Prostate cancer is the most common cancer and remains the second leading cause of cancer death in American men [1]. Despite advances in early detection and conventional treatment strategies, most patients with prostate cancer eventually progress and become resistant to treatment [2]. Several molecular pathways are involved in the progression of prostate cancer [3]. Angiogenesis, the development of new blood vessels, is recognized as one of the hallmarks of malignancy and plays a major role in tumor growth and metastasis [4-6]. Because tumor growth and metastases critically depend on the recruitment of new vessels, much effort has been expended in the development of anti-angiogenic therapies [7-13].

Endothelial cells (ECs) are the main components involved in tumor angiogenesis. Tumor angiogenesis involves capillary sprouting and abnormal branching, abnormal pericyte coat and loss of pericyte-endothelial cell adhesion, defects in the basement membrane and endothelial monolayer, and increased permeability, vasodilation and leakiness [14, 15]. An important concept in tumor angiogenesis is that tumor blood vessels contain ECs that are genetically normal and stable, which makes them less disposed to develop resistance. In contrast, tumor cells, which typically display genetic instability, are subjective to the development of resistance to therapeutic agents [6, 12, 16, 17]. However, anti-angiogenic strategies are modest in overall survival as compared with controls and the eventual development of resistance is actually quite common [18-22]. The phase III results of anti-angiogenesis therapies in prostate cancer have been disappointing [2, 18]. A double-blind, placebo-controlled phase III trial in which patients with hormone refractory prostate cancer (HRPC) and no prior chemotherapy were randomized to docetaxel with or without bevacizumab presented no demonstrable prolongation of survival with the addition of bevacizumab (median, 22.6 vs. 21.5mo) [2]. The causes of majority patients, who stop responding or do not respond at all to such drugs remain largely unexplored, despite extensive research efforts and recent advances in the understanding of this disease [23].

Because the important role of tumor-derived endothelial cells (TdECs) plays in tumor angiogenesis, study of the possible difference between TdECs and normal derived ECs (NdECs) may improve our understanding of prostate cancer biology and will lead to the development of drugs and pharmacological strategies that confer enduring anti-angiogenic therapies. Studies have shown that TdECs differ from NdECs in cell proliferation, migration, responses to growth factors and chemotherapeutic drugs [24-31]. Some studies suggest that blood vessels supplying tumors express genes not expressed in blood vessels in normal tissues [32-36]. We have previously shown that epigenetic silencing of *CYP24A1* in TdECs from a mouse syngeneic tumor model contributes to selective growth inhibition by calcitriol [37]. We further demonstrate that the *CYP24A1* promoter is differentially methylated in endothelium derived from human prostate tumor and normal lesion, indicating that epigenetic alterations in *CYP24A1* may play a role in determining the phenotype of tumor-associated vasculature in the prostate tumor microenvironment [38]. These findings indicate that identifying gene expression and/or epigenetic differences between TdECs and those in normal tissues may delineate new anti-angiogenic targets. If the molecular profile of tumor-associated vasculature is different between cancer types, identifying anti-angiogenic targets relevant to tumor types may have benefits in developing new treatment approaches [23, 39-42].

To the best of our knowledge, no information is available about global pattern of gene expression and epigenetic alterations between TdECs and NdECs in prostate cancer. In this study, we developed a method using CD31 Dynabead® positive selection and fluorescence activated cell sorting to isolate ECs from normal and malignant tissues derived from prostate surgical specimens and analyzed molecular features of the normal prostate ECs and tumor ECs from human prostate cancer.

## RESULTS

### Isolation of human normal prostate and tumor-derived endothelial cells

**Figure 1:**
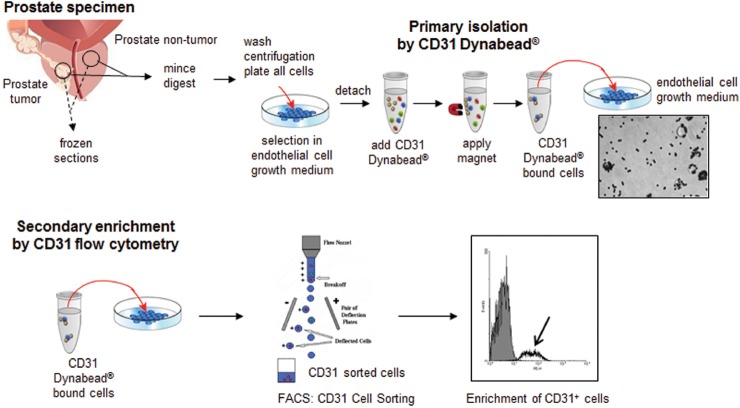
Schematic representation of prostate non-tumor and tumor endothelial cell isolation and enrichment Clinically examined and representative samples were mounted in OCT. Prostate tissues obtained from robotic radical prostectomy specimens from each patient were macrodissected for prostatic adenocarcinoma (tumor) and matched histologically benign regions. Portion of freshly macrodissected prostate tissues (tumor and non-tumor) were immediately digested and cultured in endothelial cell selection medium. Given that enough tissues were obtained, a portion of the tissues used for ECs isolation were embedded in OCT for frozen sections. ECs were isolated by CD31 magnetic Dynabead® and further enriched by CD31 fluorescent activated cell sorting (FACS).

As shown in Figure 1, prostate NdECs and TdECs were isolated using both Dynabead-based and fluorescent activated cell sorting methodologies. CD31 expression was the primary endothelial cell marker used for purification and enrichment of primary cultures of prostate NdEC and TdECs. By using the two-step Dynabead-based and FACS purification approaches, TdECs and NdECs showed >90% enrichment in primary culture.

**Figure 2:**
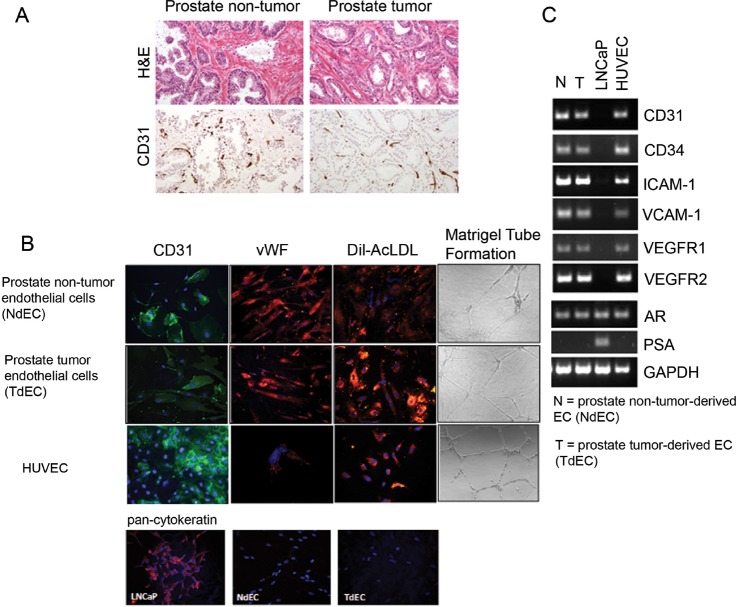
Characterization of primary cultures of endothelial cells isolated from NdECs and TdECs prostate tissue (A) Representative photomicrographs of hematoxylin and eosin stained frozen sections of benign (left) and malignant (right) prostatic tissues of macrodissected tissues used for ECs isolation. ECs are highlighted in the frozen sections by CD31 immunostaining (Original magnification, x200). (B) Representative immunofluorescent photomicrographs of CD31 (green) and vWF (red) expression and uptake of DiI-Ac-LDL (red) (original magnification, ×200) in CD31^+^ prostate TdECs and NdECs. Absence of pan-cytokeratin expression by immunofluorescence was observed in CD31^+^ prostate TdECs and NdECs. LNCaP was used as positive control for pan-cytokeratin immunofluorescence. Nuclei are stained with DAPI (blue). Endothelial tube network was formed by primary cultures of prostate TdECs and NdECs (original magnification, x100). (C) Representative reverse transcription-PCR analysis of RNA from primary cultures of TdECs and NdECs, HUVECs, or LNCaP cells using human-specific primers for human CD31, CD34, ICAM-1, VCAM-1, VEGFR1, VEGFR2, AR and PSA. GAPDH was used as a loading control.

Frozen prostate specimens obtained from robotic radical prostatectomy were evaluated by hematoxylin and eosin to ascertain regions of benign, normal- appearing prostate and regions of prostate adenocarcinoma and examined for CD31 expression (Figure 2A). Both NdECs and TdECs in primary culture demonstrated endothelial cell morphology, functionality, and marker expression profiles comparable to human umbilical vein endothelial cells (HUVECs). The cells grew in monolayers with a cobblestone morphology that was tightly associated and demonstrated clear contact inhibition.

Primary cultures of prostate NdECs and TdECs were analyzed for the expression of markers characteristic of human endothelial cells using fluorescence immunocytochemical analyses (Figure 2B). Cells were positive for endothelial cell markers by fluorescence immunostaining of human CD31 and von Willebrand Factor antigens similar to the HUVEC positive control (Figure 2B). Both NdECs and TdECs took up DiI-Ac-LDL and formed a network of capillary tubular-like structures when plated on a layer of Matrigel (Figure 2B). Cells were negative for the pan-cytokeratin epithelial cell marker by fluorescence immunostaining in contrast to LNCaP, a positive prostate adenocarcinoma epithelial cell line control (Figure 2B).

RT-PCR analyses demonstrated that cultured prostate NdECs and TdECs expressed mRNA encoding CD31, CD34, intercellular adhesion molecule-1 (ICAM-1), vascular cell adhesion molecule-1 (VCAM-1), VEGF receptor (VEGFR) 1, and VEGFR2, but were negative for PSA, consistent with the absence of secretory epithelial cells in culture. A similar expression profile was observed in HUVECs (Figure 2C). As a negative control, there was no expression of endothelial cell markers in LNCaP cells. As reported by Godoy et al, the prostate NdECs and TdECs express the androgen receptor (AR) [43]. These results indicate that primary cultures of prostate NdECs and TdECs established from clinical specimens of human normal prostate and prostate tumor were endothelial cells.

### Microarray gene profiling in TdECs and NdECs

Microarray gene expression profiling using the Affymetrix GeneChip® Human Genome U133A 2.0 was performed on 5 matched NdECs and TdECs samples (2 pairs from AA and 3 pairs from CA) and analyzed by comparing the expression profiles of TdECs to that of NdECs.

**Figure 3:**
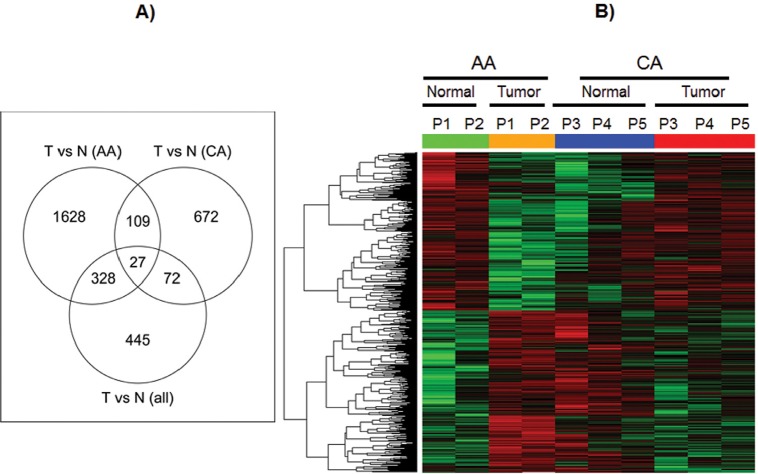
Gene expression profiles in ECs from African American and Caucasian American A) Venn diagrams showing the overlap of the differentially expressed probe sets derived from tumor vs. normal comparison from all paired samples with those from AA group only and CA group only. B) Heat map for probesets whose expression changes of tumor versus normal in AA group are significantly different from that in CA group. In heat map, red means up-regulated while green means down regulated. Green and orange bars stand for normal and tumor respectively in AA samples, while blue and red bars stand for normal and tumor respectively in CA samples.

At first, we made TdEC-versus-NdEC comparisons using all 5 pairs. As shown in Figure 3A, we identified 872 probe-sets with at least 1.5-fold differential expression at the significance level of p<0.05. Compared with the differentially expressed probe-sets derived from TdEC-versus-NdEC comparisons stratified by race, 99 of these 872 probe-sets can be derived using CA group only, while 355 probe-sets can be derived using AA group only. There are 1628, 672 and 445 probe-sets unique to the comparison using AA group only, CA group only, and all 10 samples, respectively, indicating a distinct gene expression pattern in TdECs versus NdECs from the AA and CA prostate cancer patients in this study. We identified 2,092 and 880 differentially expressed probe sets for the TdECs versus NdECs in the AA and CA groups, respectively. Only 136 probe sets are shared (Figure 3A). Hierarchical clustering of probe-sets whose expression changes in TdECs versus NdECs in AA group are found to be different from that in CA group is shown in Figure 3B.

To biologically characterize the differentially expressed genes, we performed a gene ontology (GO) enrichment analysis using the NCBI DAVID tool for the gene lists derived from the TdECs versus NdECs comparison described above. As shown in Table S1-2, pathways involved in various signal transductions, including protein kinase, extracellular matrix and cell-cell interactions, are found to be significantly enriched.

**Figure 4:**
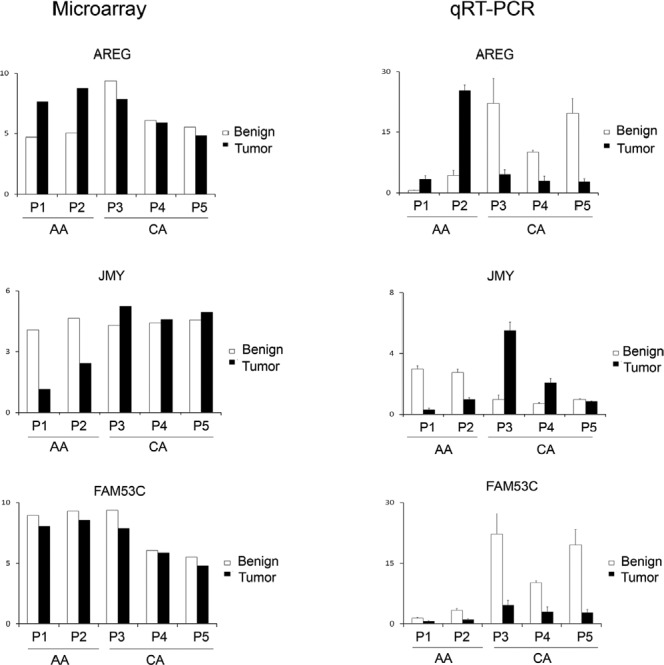
Quantitative real-time PCR validation of selected genes in endothelial cells Quantitative real-time PCR was used to validate gene expression of *AREG*, *JMY*, *EPB41*, *GMNN* and *FAM53C* in endothelial cells derived from malignant lesions vs benign lesions in AA and CA patients with prostate cancer. Relative gene expression level for qRT-PCR was normalized to the reference gene *GAPDH*. Gene expression from microarray was plotted together with the qRT-PCR results. Results were shown as Mean ± SD (triplicate). “*” represents p value <0.05 by t-Test.

To validate the gene expression level measured by microarray, we selected 3 genes, *AREG*, *JMY* and *FAM53C*, which were differentially expressed in TdECs and NdECs of AA and CA, and measured their expression level by qRT-PCR. As shown in Figure 4, there is a good agreement of expression pattern between the microarray and qRT-PCR data.

### DNA methylation profiling in TdECs and NdECs

DNA methylation profiling using the Infinium HumanMethylation450 BeadChip were performed in the same ten endothelial samples and analyzed by comparing the methylation profiles of TdECs.

**Figure 5:**
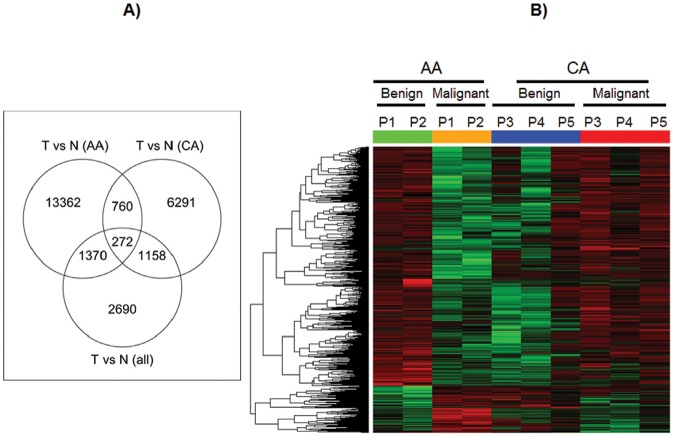
DNA methylation profile in ECs from African American and Caucasian American A) Venn diagrams Venn diagrams showing the overlap of the differentially methylated loci derived from tumor vs. normal comparison from all paired samples with those from AA group only and CA group only. B) Heat map for loci whose methylation changes of tumor versus normal in AA group are significantly different from that in CA group. In heat map, red means hypermethylated while green means hypomethylated. Green and orange bar stand for normal and tumor respectively in AA samples, while blue and red bar stand for normal and tumor respectively in CA samples.

As shown in Figure 5A, we identified 5,490 differentially methylated loci in TdEC-versus-NdEC comparisons using all 5 pairs. Compared with the differentially methylated loci derived from TdEC-versus-NdEC comparisons stratified by race, 1,430 of these 5,490 loci can be derived using CA group only, while 1,642 loci can be derived using AA group only. There are 13,362, 6,291and 2,690 loci unique to the comparison using AA group only, CA group only, and all 10 samples, respectively. We identified 15,764 differentially methylated loci in TdECs versus NdECs in the AA group, and 8,481 differentially methylated loci for the same comparison in the CA group. 1,032 loci are shared (Figure 5A). Hierarchical clustering of loci whose methylation changes in TdECs versus NdECs in the AA group are found to be different from that in CA group is shown in Figure 5B.

**Figure 6:**
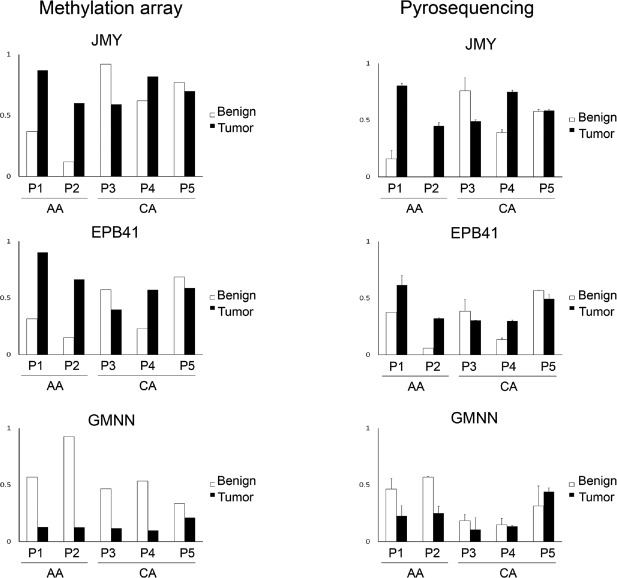
Pyrosequencing validation of selected genes in endothelial cells A) The methylation status of CpG sites detected by Infinium HumanMethylation450. B) The methylation status of CpG sites detected by pyrosequencing.

To validate the DNA methylation level measured by Illumina BeadChip, we selected 3 genes *JMY*, *EPB41* and *GMNN*, which were differentially methylated in TdECs and NdECs of AA and CA, and used bisulfite pyrosequencing to measure the methylation level at the same locus interrogated by BeadChip. As shown in Figure 6, methylation levels measured by bisulphite pyrosequencing were concordant with those estimated by BeadChip.

**Figure 7:**
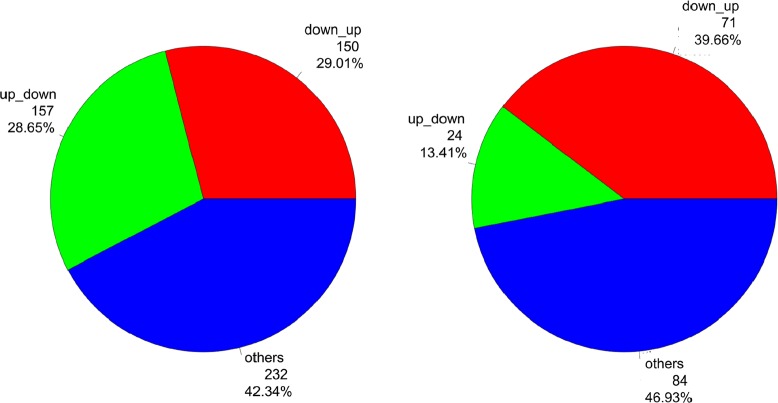
The association of differentially expressed genes with differently methylated loci Among the differentially expressed genes with differential methylation events, a portion of them have loci whose direction of methylation expression change is opposite to that of gene expression change. A) The comparison between tumor and normal in AA group. B) The comparison between tumor and normal in CA group. “up_down” indicates the genes with increased expression and hypomethylation events in tumor versus normal comparision, while “down_up” means the genes with decreased expression and hypermethylation events in tumor versus normal comparision.

To explore the interplay between expression and methylation, we examined the genes which are detected in both the expression microarray and methylation beadchip. By intercepting the differential expression results with the differential methylation results, we found 548 and 179 differentially expressed genes also have differently methylated loci in AA and CA group, respectively. A portion of them have loci whose direction of methylation expression change is opposite to that of gene expression change (Figure 7). For example, in the AA group, for the 548 differentially expressed genes with differential methylation events in the TdECs vs. NdECs comparison, 157 (29%) are up-regulated and have at least one hypomethlayed locus, while 159 (29%) are down-regulated and have at least one hypermethlayed locus. In CA group, for the 179 differentially expressed genes with differently methylated events in TdECs vs. NdECs comparison, 24 (13%) are up-regulated and have at least one hypomethlayed locus, while 71 (40%) are down-regulated and have at least one hypermethlayed locus. Gene activation might result from hypomethylation, while hypermethylation could lead to gene silencing[44]. Therefore, our data indicate the possibility that epigenetic process might partly influence the observed expression variations between TdECs and NdECs.

## DISCUSSION

Targeting angiogenesis is an important therapeutic strategy for advanced stage prostate cancer. A number of pro-angiogenic factors can induce angiogenesis. ECs are the main components involved in tumor angiogenesis. However, the difference of gene expression between NdECs and TdECs in prostate cancer remains unclear. Identification of molecules that function to regulate angiogenesis may provide us with new anti-angiogenic targets. In this study, we first established a method for the isolation of the CD31^+^ endothelial cells from tumor and normal prostate tissues obtained at prostatectomy. Both NdECs and TdECs in primary culture demonstrated endothelial cell morphology, functionality and marker expression profiles and were negative for the pan-cytokeratin epithelial cell marker. We also conducted a microarray and methylation array analysis of prostate NdEC and TdEC from prostate cancer patients, providing a pilot resource of gene expression and epigenetic regulation for these two cellular compartments in prostate cancer.

Isolation of endothelial cells from fresh benign and malignant prostate tissues by a two-step protocol including magnetic beads (Dynabeads^®^) coupled to endothelial-specific ligands and flow cytometry sorting represented a major advance in the purification of ECs from mixed-cell populations. CD31^+^ coupled magnetic beads significantly enriched EC population. Further sorting for CD31^+^ cells by flow cytometry should eliminate other possible cell contamination. TdECs and NdECs isolated by this method showed >90% enrichment in primary culture and demonstrated microvascular endothelial cell characteristics. *In vitro* primary cultures of TdECs and NdECs maintained expression of endothelial markers.

ECs from tumor blood vessels once thought to be genetically normal and stable compared to genetically instable tumor cells. However, accumulated data indicate the difference in their transcriptome between ECs derived from tumor and normal lesions [36]. ECs derived from tumor demonstrate cytogenetic abnormalities and functional abnormalities [25, 45]. The influence of the tumor microenvironment on ECs in prostate cancer was demonstrated in stimulation of HUVEC tube formation and migration by conditioned medium from LNCaP, PC-3 and DU145 prostate cancer cell lines [46].

In the present study, we observed widespread gene expression differences in endothelial cells, between malignant and normal prostate tissues. This finding is consistent with results obtained from other tumor types [36, 47].

Epigenetics of the tumor vasculature provides a new perspective on transcription control paradigms in vascular ECs and provides a molecular basis for how the environment impacts disease progression. Methylation of *GSTP1* and *RARβ2* promoters was reported in tumor-associated endothelium and stroma of localized human prostate cancer [48, 49]. Global epigenetic changes are documented for prostate cancer but not of other cells from the tumor microenvironment [47, 50, 51]. In the present study, we observed different global DNA methylation patterns in endothelial cells between malignant and normal prostate tissues. Further analysis of transcriptome and methylome data revealed a number of differentially expressed genes with loci whose methylation change is accompanied by an inverse change in gene expression.

African American men in the United States have the highest risk of developing prostate cancer, more aggressive disease, and more than twice the mortality rate observed than other racial and ethnic groups [1, 52]. The underlying reasons for this disparity are not well understood. It has been argued that the disparity may be largely due to lifestyle, dietary and socioeconomic factors, behavioral factors, socio-economic factors, gene-environment interaction, biological tumor aggressiveness and genetic factors [53, 54]. A few cDNA microarray studies have addressed different gene expression between AA and CA in prostate cancer [55-57]. However, these studies were restricted to tumor. In the present study, we compared the global gene expression and DNA methylation profiles in the endothelial cells isolated form tumor versus normal human prostate from AA and CA prostate cancer patients, respectively. We observed a wide spectrum of expression and methylation perturbation of endothelial cells between tumor and normal prostate tissues, and only a small proportion in perturbed genes overlapped between AA and CA study subjects. While preliminary and exploratory, our observations in prostate TdEC are in line with the existing studies in that group-specific alterations in tumor might exist between African-American and Caucasian-American prostate cancer patients.

Our study has strengths and limitations. The use of purified ECs largely ruled out the possible contamination from other types of cells. Despite the advantages of using purified ECs, in vitro culture may alter the behavior of cells [58]. However, previous reports showed that isolated TdECs did not lose their specific phenotype for some time after dissociation from the tumor tissue [29]. Another shortcoming of this study is the small sample size. It should be emphasized that our pilot study is exploratory in nature and the data should be interpreted with caution. Future large studies and functional experiments are necessary to confirm our observations and further explore the potential of expression and methylation perturbation to be utilized clinically as novel biomarkers for prostate cancer.

In summary, our study demonstrates the feasibility of isolation of ECs from tumor and adjacent normal prostate and further studies for human prostate cancers angiogenesis. Our study further demonstrates that identifying gene expression and/or epigenetic differences between TdECs and NdECs may provide us potential biomarkers and/or anti-angiogenic targets relevant to prostate cancer. Furthermore, data presented here provide a first glimpse of the gene expression and DNA methylation profiles in TdECs from AA and CA prostate cancer.

## MATERIAL AND METHODS

### Human prostate tissue samples

Fresh prostate tumor and matched normal tissues (n=10) were obtained from robotic radical prostatectomy specimens from men with clinically localized (organ-confined) prostate cancer treated at Roswell Park Cancer Institute (RPCI). These patients had pathologically confirmed prostate cancer and were of African-American or Caucasian-American descent by self-report. The research was approved by the RPCI Institutional Review Board. Prostate specimens free of visible cancer and prostate tumor specimens were procured as previously described [59]. Samples were excised immediately after operation. Fresh prostate tumor and matched normal tissues were placed in SPS-1® Static Preservation Solution (Organ Recovery Systems, Des Plaines, IL) on ice until EC isolation.

### Antibodies

The antibodies used for CD31 fluorescent activated cell sorting (FACS) are PE mouse anti-human CD31 (BD Pharmingen, San Diego, CA) and PE mouse IgG1 κ isotype control (BD Pharmingen). Primary antibodies for cell fluorescent staining and flow cytometric analysis of human prostate NdECs and TdECs are: rabbit anti-human von Willebrand factor (Dako, Carpinteria, CA), mouse anti-human cytokeratin MNF116 (Dako), and mouse anti-human CD31 (JC70A, Dako). Goat serum and donkey serum used for blocking were obtained from Sigma. 4',6-diamidino-2-phenylindole (DAPI) was obtained from EMD Chemicals (Gibbstown, NJ).

### Cell cultures

Primary cultures of human prostate endothelial cells (NdECs and TdECs) were cultured in endothelial growth medium [Endothelial Cell Growth Medium MV2 with Supplement Mix (PromoCell GmbH, Heidelberg, Germany) and 100 U/ml penicillin and 100 μg/ml streptomycin (Invitrogen)]. HUVECs were obtained from Lonza (Walkersville, MD) and maintained under the same conditions. LNCaP prostate adenocarcinoma cell line was obtained from American Type Culture Collection (ATCC, Manassas, VA) and cultured under conditions specified by ATCC.

### Isolation and primary culture of prostate endothelial cells

The multiple steps in prostate NdECs and TdECs isolation and enrichment are shown in Figure 1. Briefly, excised human prostate tumor and non-tumor tissues in SPS-1® were minced into 2 mm^3^ pieces and digested for 16 h at 4°C with dispase (2.4 U/ml) (Invitrogen, Carlsbad, CA) in 1× DPBS Mg^2+^/Ca^2+^-free. Undigested tissues after dispase digestion was further digested in a digestion mixture consisting of 0.28% collagenase, type II (Sigma, St. Louis, MO), 2.4 U/ml dispase, 0.01% DNase I (Sigma) in 1X DPBS Mg^2+^/Ca^2+^-free (Invitrogen) for an additional hour at 37°C. Cell suspensions were filtered sequentially through 100 μm and 40 μm cell strainers to remove undigested tissue. The heterogeneous cell suspensions were washed twice with endothelial growth medium, plated onto 10 μg/cm^2^ collagen, type I from calf skin (Sigma, St. Louis, MO) coated 25-cm^2^ flasks, and selectively grown in endothelial growth medium (Figure 1) for approximately 1-2 weeks at 37°C, 5% CO_2_. At 80-90% confluence, human prostate ECs were isolated using the Dynal MPC®-L Magnetic Particle Concentrator (Invitrogen Dynal AS, Oslo, Norway) and anti-CD31 conjugated magnetic Dynabeads^®^ (Invitrogen Dynal AS) according to the manufacture's protocols. Concentrated CD31-positive ECs were re-plated and grown in collagen-coated flasks with endothelial growth medium. After subculture for ~2 weeks, bead sorted CD31-positive ECs were further enriched by a second sort using CD31 fluorescent activated cell sorting (FACS). PE-CD31 labeled ECs were resuspended in 500 μl FACS Buffer and filtered prior to cell sorting on the FACSAria I cell sorter and processed on the FACSDiva software (Becton-Dickinson, San Jose, CA). Recovered CD31-positive cells were grown in collagen-coated flask with endothelial growth medium for no more than six passages for experimental studies.

### Immunohistochemical and immunofluorescence analysis

Hematoxylin and eosin (H&E) stained sections were obtained from OCT frozen samples of specimen used for EC isolation. For immunofluorescent staining of TdECs, NdECs, or HUVECs, cells were seeded at 1×10^4^ cells per well of a 24-well plate containing collagen-coated glass coverslips. After cells attached, they were fixed in situ with 4% paraformaldehyde at 37°C for 30 min. Thereafter, cells were washed 3 times with 1× DPBS for 5 min each. Cells were permeabilized with 0.1% Triton X-100 in 1× DPBS at 37°C for 15 min, washed 3 times with 1x DPBS for 5 min each, blocked with 1:100 dilution of donkey or goat serum at 37°C for 1 hr, then washed again, and incubated with 400 μl primary antibody in 1% BSA in 1× DPBS overnight at 4°C. Primary antibodies were used at 1:1000 for mouse anti-human CD31 (JC70A; Dako), 1:400 for rabbit anti-human von Willebrand factor (Dako) and 1:100 for mouse anti-human cytokeratin (Dako). After washes, fluorochrome-conjugated secondary antibody in 1% BSA in 1× DPBS was applied and incubated for 1 hr at 37°C. Secondary antibodies were used at 1:3000 for Cy2 donkey anti-mouse (CD31), 1:3000 for Cy3 goat anti-rabbit (vWF), and 1:400 for AlexaFluor 594 goat anti-mouse (cytokeratin). Thereafter, the stained cells were washed with 1× DPBS three times for 15 min each. Nuclei were stained with 300 nM DAPI solution. Coverslips with cells were mounted on glass with Fluoromount gel (Sigma) and dried for ~1 hr at 4°C. Fluorescence was observed with an Olympus BX40 Trinocular Fluorescence Microscope (Olympus America Inc., Melville, NY) and captured using a QImaging camera and QCapture Pro software (QImaging, Surrey, BC, Canada).

### Uptake of DiI-Ac-LDL

The presence of scavenger receptors for acetylated low density lipoprotein (ac-LDL) on NdECs and TdECs was detected using 1,1'-dioctadecyl-3,3,3',3'-tetramethyl-indocarbocyanine perchlorate acetylated LDL (DiI-Ac-LDL; Invitrogen Molecular Probes, Carlsbad, CA). ECs were plated cells at a density of 5×10^4^ − 1×10^5^ cells/ well in a 24-well plate containing collagen-coated glass coverslips and grown in endothelial growth medium for 24 h. ECs were incubated with 10 μg/ml DiI-Ac-LDL for 4 h at 37°C in 5% CO2 according to the published method [60]. After incubation, cells were washed with 1x DPBS to remove excess DiI-AcLDL and fixed with 10% buffered formalin phosphate for 5 min. Coverslips containing cells were mounted on slides with VECTASHIELD® Hard-Set™ Mounting Medium containing 4',6-diamidino-2-phenylindole (DAPI;Vector Laboratories Inc., Burlingame, CA). LDL uptake was examined using an Olympus BX40 with an optical filter set with an excitation of 545 nm and emission of 590 nm and captured using a QImaging camera and QCapture Pro software.

### Endothelial cell tube formation on Matrigel

The ability of prostate NdECs and TdECs to form capillary-like structures was evaluated by placing them on a solubilized basement membrane preparation Matrigel (Becton-Dickinson), according to the manufacturer's instructions. Matrigel (1 mg/ml) was thawed overnight at 4°C. Pre-cooled 24-well plates were coated with 250 μl Matrigel using chilled pipettes. Matrigel-coated plates were incubated at 37°C for 30 min. ECs were seeded onto Matrigel-coated wells at a density of 5×10^4^ cells/well in endothelial growth medium and incubated for 16 h at 37°C in 5% CO_2_. HUVECs were used as a positive control for tube formation. Capillary tube-like structures formed by ECs were washed once with 1x DPBS, fixed with 4% paraformaldehyde, pH 7.4 at 37°C for 15 min, and kept hydrated in 1× DPBS. Capillary tube-like structure formation was assessed microscopically using an Olympus IMT-2 inverted microscope (Olympus America Inc., Center Valley, PA) equipped with SPOT RT Slider camera and a SPOT Advanced imaging software (Diagnostic Instruments, Inc., Sterling Heights, MI).

### RNA extraction

RNA was extracted from ECs using RNeasy mini kit according to manufacturer's instruction (Qiagen, Valencia, CA).

### Affymetrix human U133 plus 2.0 array

We used the Bioconductor packages in the R statistical computing environment for Affymetrix Human U133 plus 2.0 array data processing. Specifically, the MAS5.0 function was used to generate expression summary values, followed by trimmed mean global normalization to bring the mean expression values of all ten arrays to the same scale. We filtered out probe sets whose expression-status was called absent (i.e., indistinguishable from the background intensity) across the samples. We used the Limma program in Bioconductor package to calculate the statistical significance for the level of differential expression. Briefly, a linear model was fit to the data, with cell means corresponding to the different conditions, and a random effect for array. The differential expression is set with at least 1.5-fold expression change at P value < 0.05.

### Quantitative real-time PCR (qRT-PCR)

Total RNA (500 ng) was harvested from ECs and converted to cDNA using oligo primers in a final volume of 20 μl using first strand cDNA synthesis kit according to manufacturer's instruction (Roche, Indianapolis, IN). Two μl of cDNA was used for qRT-PCR in 20 μl-reactions to determine the relative expression of target genes using a TaqMan-based real-time PCR and a 7300 Real-Time PCR System (Applied Biosystems). TaqMan® Gene expression assays (primers and probe) were obtained from Applied Biosystems. All assays were performed using triplicate samples for each cDNA synthesis. Data was analyzed using the RQ study software from 7300 Sequence Detection System package. Gene expression for all transcripts was normalized to the endogenous control gene human *GAPDH*.

### DNA methylation analysis

Methylation data were assembled with GenomeStudio methylation software from Illumina. The methylation level (β value) generated from GenomeStudio software ranged between (0, 1). 0 indicates absent methylation and 1 indicates complete methylation. Raw average β values were analyzed without normalization as recommended by Illumina. For data quality control, we excluded the CpG loci with missing β values, loci which are on the X chromosome, and loci with a median detection P value greater than 0.05 across the samples. The level of differential methylation was estimated using linear model with factorial design implemented in Limma. The differential methylation is set with at least 0.05 β value changes at P value < 0.05.

### Bisulfite DNA pyrosequencing

DNA was extracted from TdECs and NdECs cell samples using the Qiagen DNA purification kit (Qiagen). Bisulfite treatment was carried out using the EZ DNA Methylation-Gold Kit (Zymo Research, Irvine, CA) according to the manufacturer's instructions. PCR amplification was carried out using Faststart Taq Polymerase (Roche) and the following cycle conditions: 95 °C for 3 min, then 45 cycles of 95°C for 30 sec, 52°C for 30 sec and 72 °C for 30 sec. Quantitative pyrosequencing of bisulfite PCR products was carried out using the PSQ 96 Pyrosequencing System with Pyromark Q96 MD platform (Biotage, Charlotte, NC).

## Supplementary Tables


